# A Rare Presentation of Myoepithelioma of the Parotid Gland Manifesting as an Infra-Auricular Swelling

**DOI:** 10.7759/cureus.70746

**Published:** 2024-10-03

**Authors:** Yash Kalra, Saahiti Koppolu, Manu Babu, Rashmi Prashant, Akshi Raj, Mayur Ingale

**Affiliations:** 1 Department of Otolaryngology, Head and Neck Surgery, Dr. D. Y. Patil Medical College, Hospital and Research Centre, Dr. D. Y. Patil Vidyapeeth (Deemed to be University), Pune, IND; 2 Department of Pathology, Dr. D. Y. Patil Medical College, Hospital and Research Centre, Dr. D. Y. Patil Vidyapeeth (Deemed to be University), Pune, IND

**Keywords:** benign parotid tumors, myoepithelioma, parotid myoepithelioma, rare case report, unusual presentation

## Abstract

This report describes an uncommon tumor of the salivary glands, myoepithelioma, that primarily affects major and minor glands, with a notable predilection for the parotid gland. Typically benign, this tumor arises from aberrant myoepithelial cells situated between the basement membrane and acinar cells. Myoepitheliomas are considered a subset of pleomorphic adenomas, distinguished by excessive myoepithelial cell growth. Despite their initial discovery, the precise histopathological and immunohistochemical characteristics of these tumors remain elusive, posing a diagnostic challenge because of their complex nature.

We discuss a case of a 42-year-old female who had a 2 x 2 cm lump in the right infra-auricular area. The lump was examined with ultrasonography (USG) and later surgically removed. The initial frozen section analysis indicated an oncocytic lesion, but further histopathological and immunohistochemical evaluations confirmed that it was a myoepithelioma of the parotid gland.

## Introduction

Myoepithelial cells, found in salivary gland acini and intercalated ducts, exhibit a unique blend of epithelial and smooth muscle cell characteristics. Myoepithelioma is a rare type of salivary gland tumor, accounting for only about 1% of all salivary gland neoplasms. Although it is usually benign and found in major salivary glands, it can also appear in minor glands, such as those in the palate, and may occasionally show malignant characteristics. Although these tumors are typically benign and tend to recur only when surgical resection is incomplete, a few instances of malignant forms have been reported as well, with an incidence of 0.4-0.6% of all salivary gland neoplasms [1]. This tumor can affect individuals of any age or gender and typically presents as a slow-growing, painless mass, commonly located in the parotid gland [2]. Myoepitheliomas have also been identified in a variety of other sites, such as the breast, nasopharynx, retroperitoneum, larynx, lung, and skin [3].

These tumors generally expand locally without infiltrating surrounding structures like the facial nerve, which distinguishes them from other parotid masses. The diagnosis involves radiologic imaging and histological examination, with surgical excision being the preferred treatment. However, the role of chemoradiation is unclear due to the tumor's rarity. Recurrence rates range from 15 to 18%, with potential for malignant transformation linked to c-kit receptor overexpression and p53 mutations. Additionally, myoepithelial carcinoma can develop de novo. Carcinomas account for roughly 10% of all myoepithelial neoplasms [4].

We present a case of a 42-year-old female diagnosed with a myoepithelioma of the right parotid gland, highlighting the need for precise diagnosis and appropriate treatment.

## Case presentation

A 42-year-old woman presented with a two-month history of swelling in the right infra-auricular area, gradually increasing in size from that of a peanut to a lemon. She experienced intermittent pain in the region and right ear, which had been worsening over the past 15 days. There was no reported discharge from the swelling, facial asymmetry, dryness of mouth, or fever.

**Figure 1:**
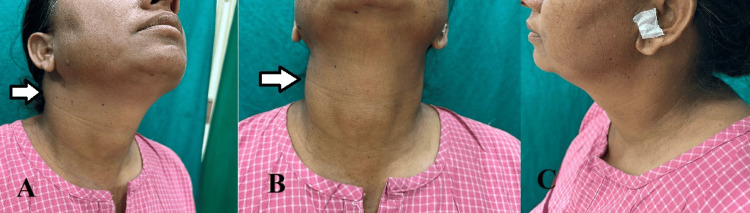
Preoperative images showing the swelling on the right lateral aspect of neck A: Right lateral profile with the arrow denoting the swelling. B: Central profile with the arrow denoting the swelling. C: Left lateral profile

Clinical examination revealed a solitary, firm, smooth, and mobile swelling (2 x 2 cm) in the right infra-auricular region, free from the overlying skin with no local rise of temperature (Figure 1). It extended from 2 cm below the mastoid tip to 10 cm above the clavicle (superior to inferior) and from the angle of the mandible to the anterior border of the right sternocleidomastoid muscle (medial to lateral). No other significant findings were noted. There was no facial nerve palsy, no growth or lump in the oropharynx, and no xerostomia. There was no cervical lymphadenopathy and no other salivary glands were palpable. The remainder of the otorhinolaryngological examination was normal. A provisional diagnosis of a benign parotid tumor in the right superficial lobe was made with a differential diagnosis of myoepithelioma, pleomorphic adenoma, oncocytoma, or Warthin's tumor.

Further investigations showed normal blood parameters, except for mildly low levels of hemoglobin (Table 1).

**Table 1:** Blood parameters HBsAg: hepatitis B surface antigen; HCV: hepatitis C virus; HIV: human immunodeficiency virus; S/CO: single to-cut-off ratio

Variable	Patient value	Reference value
Hemoglobin (g/dL)	10	11.6-15.0
Total leucocyte count (/µL)	8800	4000-10,000
Platelet count (/µL)	402,000	150,000-410,000
Absolute neutrophils (/µL)	5808	2000-7000
Absolute eosinophils (/µL)	176	20-500
Absolute lymphocytes (/µL)	2376	1000-3000
Erythrocyte sedimentation rate (mm/hour)	15	Upto 20
HIV (I and II) (S/CO)	Nonreactive (0.06)	<1.00
HBsAg (IU/mL)	Nonreactive (0.001)	<0.05
HCV (S/CO)	Nonreactive (0.08)	<1.00
Urea (mg/dL)	17	17-49
Creatinine (mg/dL)	0.62	0.6-1.35
Blood group	O positive	-

Ultrasonography (USG) revealed a well-defined, heterogeneously hypoechoic cystic lesion (20 x 21 x 28 mm) with a solid component within, measuring 7 x 9 x 8 mm, and multiple free-floating internal echoes and thick septations within the right parotid gland superficial lobe. The solid component showed internal vascularity. A few subcentimetric cervical lymph nodes were noted at levels two and three bilaterally.

**Figure 2:**
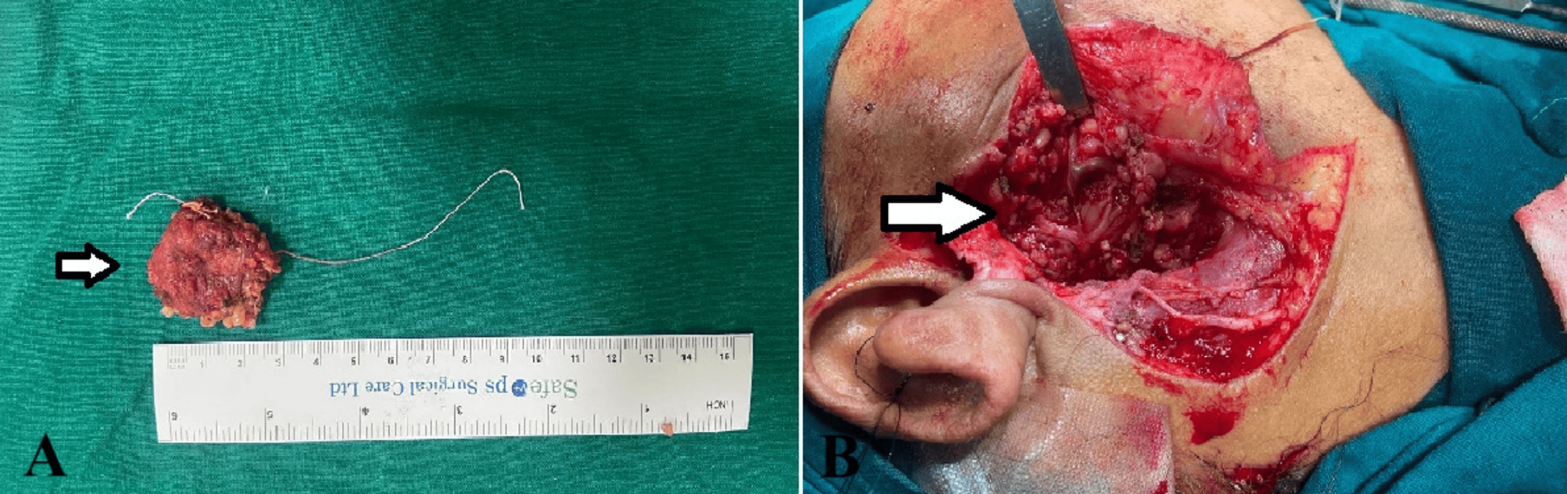
Intraoperative images A: White arrow denoting the excised specimen sent for histopathological examination. B: White arrow denoting the right parotid region post-excision

A right superficial parotidectomy with a modified Blair incision was performed under general anesthesia, preserving all facial nerve branches after identifying the posterior belly of the digastric muscle, which lies inferior to the facial nerve and identifying the tragal pointer. The facial nerve lies medial and about 1 cm inferior to the tragal pointer (Figure 2).

**Figure 3:**
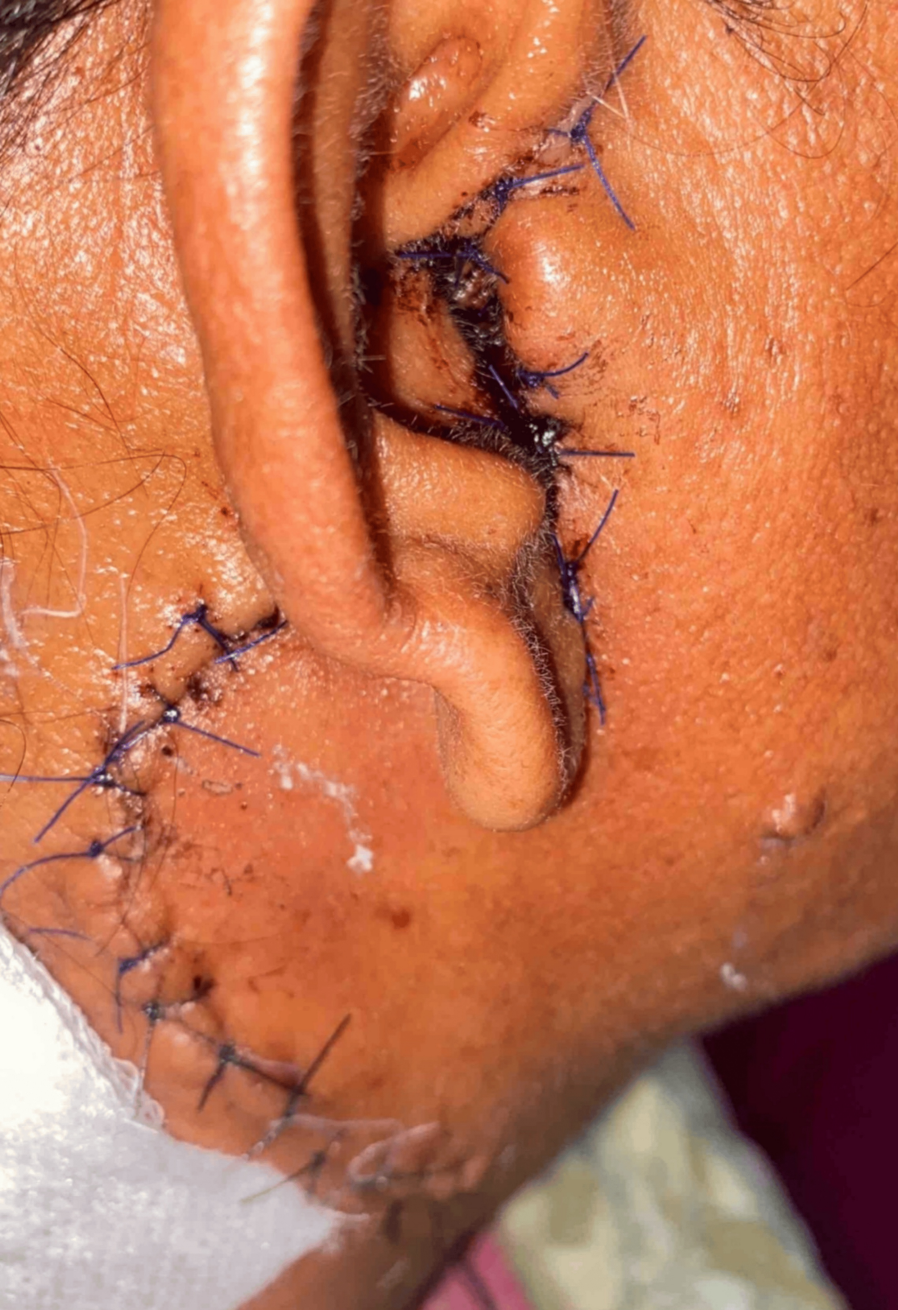
Postoperative image showing the surgical site closed with prolene sutures

Intraoperative frozen sections suggested a benign cystic oncocytic lesion with chronic sialadenitis with no evidence of any obvious invasion. The surgical site was then closed in two layers (Figure 3). Post-surgery, the patient experienced an uncomplicated recovery, without any facial nerve issues or other problems.

**Figure 4:**
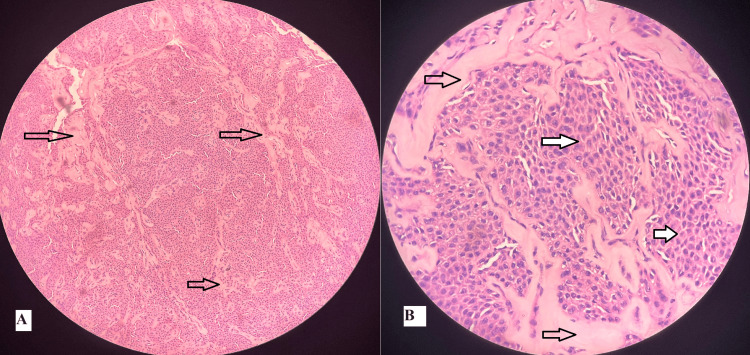
Histopathological images of the excised specimen A: Hematoxylin and eosin stain, 100x magnification: the whole field shows cells arranged in sheets with the arrows marking eosinophilic acellular areas in the stroma. B: Hematoxylin and eosin stain, 400x magnification: black arrows without fill marking eosinophilic acellular areas in stroma; white solid fill arrows marking round to polygonal cells with central to eccentric nuclei and eosinophilic granular cytoplasm

Final hematoxylin and eosin-stained sections showed a fairly circumscribed lesion composed of round to polygonal cells, central to eccentric nuclei, inconspicuous nucleoli, as well as a moderate amount of eosinophilic granular cytoplasm arranged in sheets. Stroma showed eosinophilic acellular areas. Sections from the cyst wall showed the lining epithelium of oncocytic cells. There was no evidence of mitosis, necrosis, atypia, or malignancy in the sections (Figure 4). On immunohistochemistry examination, CK7 and p63 were found to be positive, while S100 was found to be negative. Therefore, the final histopathological and immunohistochemistry findings favored a diagnosis of myoepithelioma.

After the surgery, the patient experienced no complications with wound healing and achieved full recovery within a month. The patient continues to have regular follow-up appointments with no evidence of recurrence. The patient has been advised to have regular follow-ups every three months for one year.

## Discussion

Myoepitheliomas are infrequent, accounting for fewer than 1.5% of tumors of the salivary glands. They most frequently occur in the parotid gland (40%) and palate (21%), and do not have specific clinical characteristics. These tumors usually appear as slow-growing, asymptomatic masses, much like many other salivary gland tumors. The age and gender distribution for myoepitheliomas mirrors that of mixed tumors, which can make diagnosis difficult [5].

Myoepithelial cells, derived from ectoderm, possess epithelial characteristics. According to Line and Archer, terminal tubule epithelial cells may serve as precursor cells [6]. The morphology of myoepithelial cells varies, with stellate shapes in acinar positions and bipolar shapes near ductal elements. Beyond salivary glands, myoepithelial cells are found in sweat glands, mammary glands, Bartholin’s glands, and tracheal and esophageal mucus-secreting glands. They are also found in the prostate and lacrimal glands, showcasing their widespread presence in various tissues [7].

The pathological definition of myoepithelioma remains a topic of debate due to the diverse morphological appearances of neoplastic myoepithelial cells. While most myoepitheliomas are biologically benign, they can display local invasive behavior and metastatic potential in rare cases. Histological examination of salivary gland-derived myoepitheliomas reveals three primary cell types: epithelioid, spindle, and plasmacytoid. Notably, epithelioid cells may occasionally exhibit clear cytoplasmic features, adding to the complexity of diagnosis [8].

Myoepitheliomas have been identified in various extrasalivary gland locations, including the breast, larynx, and skin. These tumors exhibit similar morphological and immunohistochemical characteristics to their salivary gland counterparts. Pleomorphic adenomas or benign myoepitheliomas can give rise to myoepithelial carcinoma. Distinguishing between benign and malignant myoepitheliomas relies on histological features such as pleomorphism, atypia, increased mitotic rate, necrosis, and invasive growth patterns. The consistent expression of CK in myoepitheliomas makes its absence a strong argument against this diagnosis. Vimentin and S-100 protein, typically absent in normal myoepithelial cells, serve as sensitive markers for neoplastic myoepithelium. However, smooth-muscle differentiation markers are less useful due to the potential loss or alteration of the smooth-muscle phenotype during neoplastic transformation [9].

To identify a tumor as a pure myoepithelioma, the epithelial component must be less than 5-10% of the total tumor, and it should not contain fibromyxoid stroma. The presence of such stroma usually points to a pleomorphic adenoma. However, some experts argue that even minor epithelial differentiation could lead to a diagnosis of pleomorphic adenoma, underscoring the challenges in differentiating these tumors [10].

When diagnosing plasmacytoid hyaline myoepithelioma, it is important to differentiate it from plasmacytoma, rhabdomyoma, and oncocytoma. The unique hyaline cytoplasm of myoepithelioma distinguishes it from the granular cytoplasm found in oncocytoma. Clear cell myoepithelioma must be differentiated from renal cell carcinoma metastases, clear cell salivary gland carcinomas, and other salivary gland tumors with clear cell features. Similarly, spindle cell myoepithelioma can be mistaken for nerve sheath tumors, fibrohistiocytic tumors, nodular fasciitis, or synovial sarcoma due to their similar morphological characteristics [11].

Diagnostic methods for myoepithelioma include USG, fine-needle aspiration cytology (FNAC), CT, and MRI. However, the final histopathological report of the excised specimen is often necessary for a definitive diagnosis. On CT scans, parotid gland myoepitheliomas typically appear as well-defined, smooth, or lobulated masses with homogeneous or heterogeneous enhancement [12]. Myoepitheliomas exhibit diverse morphological patterns, including spindle, plasmacytoid, epithelioid, and clear cell types, making pre-surgical diagnosis via FNAC challenging. Histopathological examination and immunohistochemical staining of the resected specimen are crucial for diagnosis, with neoplastic myoepithelial cells typically expressing cytokeratin, vimentin, S-100 protein, calponin, smooth muscle actin (SMA), and glial fibrillary acidic protein while being negative for carcinoembryonic antigen [13].

The main treatment for myoepithelial carcinomas is surgical excision, with a focus on achieving wide margins to minimize the high recurrence risk. Adjuvant chemotherapy and radiotherapy have not shown effectiveness in managing these carcinomas [5]. Myoepitheliomas generally have a benign course and exhibit low recurrence rates. Treatment should follow the guidelines for benign salivary gland tumors, which involve superficial parotidectomy for a parotid tumor and submandibular gland excision for a submandibular gland tumor. The recurrence rate for benign myoepitheliomas is comparable to that of pleomorphic adenomas (15-18%). Regular follow-up examinations are crucial to monitor for potential local recurrence, and the overall prognosis for benign myoepitheliomas is favorable.

## Conclusions

This case report provides an in-depth analysis of myoepithelioma, a biologically benign salivary gland lesion. Due to the lack of distinctive clinical features, its accurate diagnosis relies on immunohistochemical studies to differentiate it from other parotid tumors. This tumor must be considered in the evaluation of a parotid mass to ensure an accurate diagnosis. Following diagnosis, surgical excision in the form of superficial parotidectomy is the primary treatment approach. In our case, a superficial parotidectomy was performed to remove the tumor while preserving the facial nerve, resulting in an uneventful postoperative period. Further research is required to investigate the role of imaging techniques like CT and MRI in diagnosing benign parotid tumors and to develop less invasive treatment options.
